# Multifunctional Nanoscale Pigments: Emerging Risks and Circular Strategies for a Sustainable Future

**DOI:** 10.1002/smsc.202500240

**Published:** 2025-09-29

**Authors:** Ajay Vikram Singh, Preeti Bhardwaj, Vimal Kishore, Sunil Choudhary, Akihiko Hirose, Neha Gupta, Madleen Busse, Swarn Lata Singh, Christopher J. Osgood

**Affiliations:** ^1^ Department of Chemical and Product Safety German Federal Institute for Risk Assessment (BfR) Max‐Dohrn‐Straße 8‐10 Berlin 10589 Germany; ^2^ Department of Physics Banaras Hindu University Varanasi 221005 India; ^3^ Department of Radiotherapy and Radiation Medicine Institute of Medical Sciences Banaras Hindu University Varanasi 221005 India; ^4^ Chemicals Assessment and Research Center Chemicals Evaluation and Research Institute Tokyo 112004 Japan; ^5^ Department of Radiation Oncology Apex Hospital Varanasi 221005 India; ^6^ Department of Biological Safety German Federal Institute for Risk Assessment (BfR) Berlin 12277 Germany; ^7^ Department of Physics Mahila Mahavidyalaya (MMV) Banaras Hindu University Varanasi 221005 India; ^8^ Department of Biological Sciences Old Dominion University Norfolk 23529 VA USA

**Keywords:** emerging contaminants, environmental sustainability, green chemistry innovations, interdisciplinary risk assessments, nanoscale pigments, One Health frameworks

## Abstract

The substantial penetration of nanoscale pigments into a range of sectors has changed the dynamics of industries such as medical, material science, and many more. Nonetheless, their persistence in the environment and probable adverse impacts on health require that an assessment of such risks be formulated considering the One Health perspective. This viewpoint considers the crossing of boundaries of progress in the nanotechnology of nanoscale pigments with environmental, animal, and human health and emphasizes the significance of collaborative activity. Traditional perspectives explain the distribution of pigment history, while the nanotechnology of today's accessibility poses problems regarding utilization, toxicities, and interactions with the environment. Through discussions of environmental pathways, health determinants, and regulatory insufficiencies, this work makes evident that pigments are critical both as emerging contaminant's and as innovation drivers. The necessary advancements in exposure minimization and sustainable practices are discussed as well, giving insight on benign‐by‐design techniques and circular economy solutions. Expanding the discussion of the existing knowledge and the gap where the ´One Health´ concept can be applied in physiochemical properties of pigments as well as governance, this work offers an approach to enabling risk while enhancing invention. It urges timely global action for sustainable, beneficial nanoscale pigment futures.

## Graphical abstract

The growing use of nanoscale pigments drives innovation across sectors, yet raises environmental and health concerns. This article explores their multifunctional roles, emerging risks, and regulatory gaps through a One Health lens. It highlights sustainable strategies—including benign‐by‐design approaches and circular economy principles—crucial for balancing technological advancement with ecological and human safety.

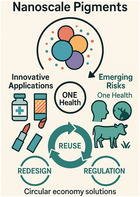

## 1 Introduction

Over 140 000 chemicals have been produced and heavily used since the 1950s, yet only ≈5000 are estimated to pose a global threat through widespread environmental dispersion, highlighting the critical role of vast data resources like PubChem, which contains 96 million entries, in ONE HEALTH concept‐based exposomics.^[^ ^1^ ^]^ Pigments are chemicals widely used in industries such as textiles, cosmetics, and food for their aesthetic and functional properties, and organic pigments—unlike inorganic ones—are water‐insoluble and inherently remain at the nanoscale, where recent nanotechnology advances have further enhanced their optical, thermal, and chemical performance. These nanoscale pigments offer novel features like high surface area, new reactivity, and superior dispersibility, which can be advantageous across several different applications. But those same properties may also be responsible for their unexpected impacts, triggering environmental, and health impacts, raising concerns about their production, usage, and eventual disposal.^[^ ^2^ ^]^ One Health perspectives provide a more comprehensive comparison of nanoscale pigment risks and benefits. In the environment and in living systems, pigments, like other emerging contaminants, are capable of crossing pathways, which allows for a multitude of interactions and possible bioaccumulation.^[^ ^3^ ^]^ A transition from traditional to nanoscale pigments is part of a larger industrial shift to chemical entities of new types, which can be seen in the large scale way, with which new chemicals are introduced into global supply networks.

This perspective article highlights the urgent need to address emerging contaminants, such as pigment, through crossdisciplinary approaches. Despite global efforts to regulate conventional pollutants, the scale and complexity of nanoscale entities require modern monitoring, remediation, and policy integration strategies.^[^ ^4^ ^]^

In this perspective, we propose a viewpoint on nanoscale pigments using One Health approach, define the properties of nanoscale pigments, their environmental pathways and health implications, as well as their potential sustainable practices for management. The article adds to the increasing volume of knowledge on emerging contaminants, positioning nanoscale pigments as a challenge and opportunity with respect to One Health.

## 2 Historical Context of Pigments in Environmental and Health Sciences

**Figure 1:**
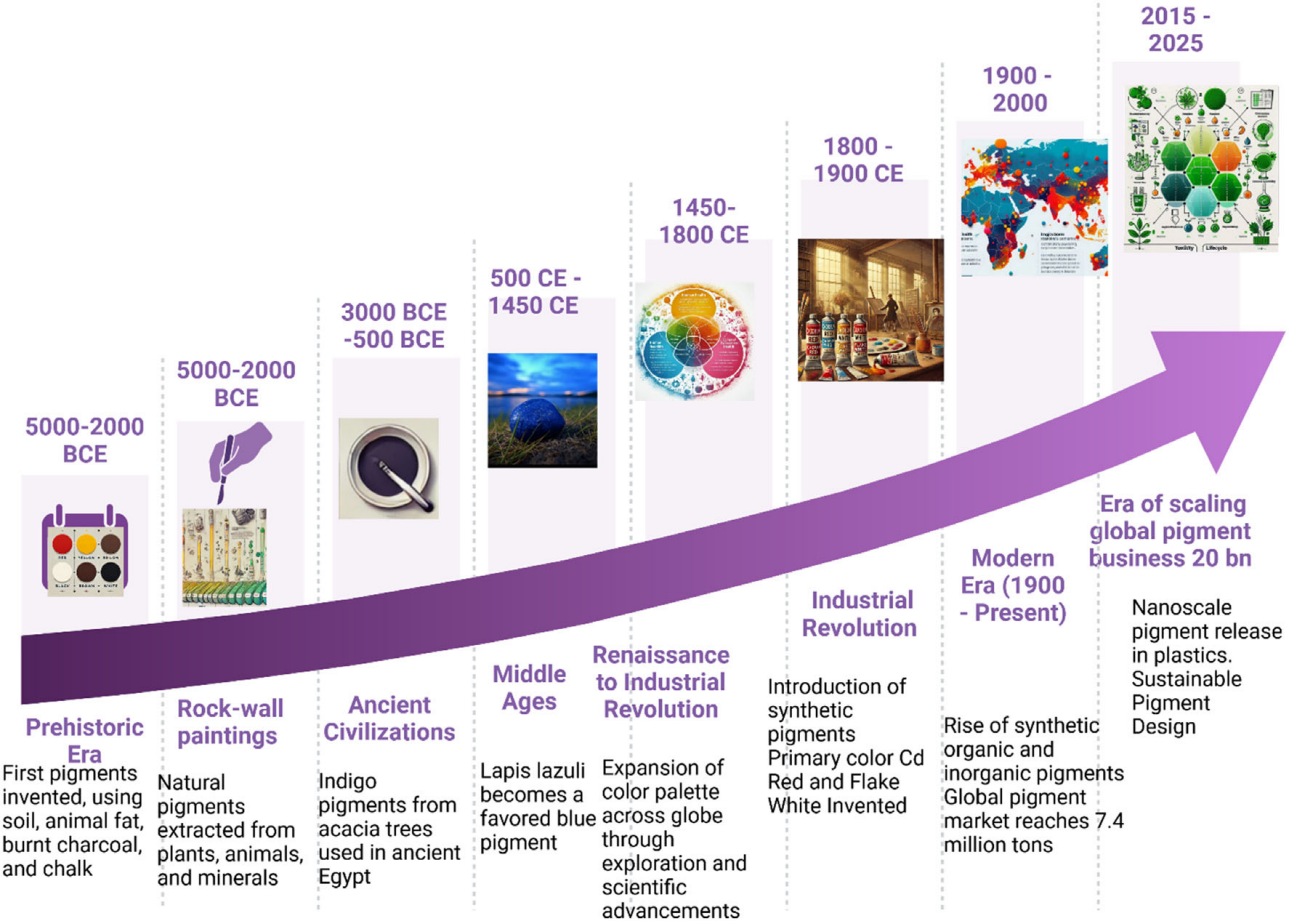
Evolution of pigment use and environmental impact. A timeline of pigment applications and their environmental impacts from prehistoric times to the present illustrates the evolution of pigments and their environmental impacts. Using the *y*‐axis, you can view the timeline in years, while using the x‐axis to categorize pigment usage in art, medicine, and industry. Important milestones include Prehistoric Era (40 000–3000 BCE): Natural pigments based on Earth, charcoal, and minerals. Minimal environmental impact. Ancient Civilizations (3000 BCE–500 CE): The emergence of synthetic pigments, such as Egyptian blue. Broader use in art and in early medicine. Middle Ages (500–1450 CE): New mineral pigments are introduced. Few uses in industry. Renaissance to Industrial Revolution (1450–1800): Wider range of color palette. Higher usage in art and in early industrial processes. Industrial Revolution (1800–1900): Invention of synthetic pigments. Increase in environmental awareness as a result of industrial rate. Modern Era (1900–Present): Nanoscale pigments evolution. Broader usage in art, medicine, and industry. An increasing awareness of their environmental and health impacts. Future Outlook: Transitioning to greener practices and green chemistry for pigment production (image drawn with Biorender.com).

Pigments have been important to humans throughout history, from naturally occurring substances like ochres and plant extracts to synthetics developed during the industrial revolution. This spectrum of development in pigments, their production, and applications exemplifies the significant growth in Industrial Chemistry and technology, notably in chemical synthesis and materials engineering as depicted in **Figure** **1**. Though classic pigments were majorly born for decorative or artistic applications, their use was diversified by industrialization to textiles, paints, cosmetics, and advanced materials.^[^ ^5^ ^]^

### 2.1 Industrialization and the Emergence of Synthetic Pigments

The manufacture of synthetic pigments—including both inorganic and organic compounds—became possible with the advent of industrial‐scale pigment production in the 19th and 20th centuries. Great improvements were made in color vibrancy, durability, and cost using these early pigments (Figure 1 time line). But as they became ubiquitous, they too raised social and health concerns. We acknowledge that legacy contaminants such as PAAs, PAHs including intentionally added lead and cadmium pigments are now verified as toxic to humans and ecosystems; therefore, bans or restrictions are being placed in many regions.^[^ ^6^ ^]^

### 2.2 Transition to Nanoscale Pigments

The second chemical revolution began in the mid‐20th century with the advancement of nanoscale materials such as pigments. This transition is consistent with the advancements of nanotechnology that led to the development of pigments with enhanced optical and functional properties. These nanoscale pigments are increasingly being incorporated into systems that allow for high‐performance material designs for applications in photonics, energy storage, and biomedical imaging.^[^ ^7^ ^]^ While useful, the environmental and health effects of nanoscale pigments are understood too little and remind of potential emerging contaminating (EC) facets. Such issues highlight the need for examining historical trends of pigment usage in order to better understand modern‐day patterns for sustainable practices in pigment utilization. **Table** **1** identifies important knowledge gaps, their implications for One Health, and targeted solutions across the environmental, health, regulatory, and innovation domains in order to methodically address the fragmented understanding of nanoscale pigments.^[^ ^8^, ^9^, ^10^ ^]^

**Table 1:** Evolving “unknowns” in risk assessment of nanoscale pigments within the One Health agenda.

Category	Unknown Facts	Implications for One Health	Proposed Solutions
Environmental Pathways	‐Little understanding of degradation of pigments in different environmental matrices (soil, water, air). ‐Extent of bioaccumulation of pigments in food chains unexplored.	‐Persistent pigments can build up in the environment, posing long‐lasting risks. ‐Pigments accumulated in biological bodies may be transferred to the diets of both humans and animals, resulting in health risks.	‐Experimental studies on history and conditions of pigment degradation kinetics and by‐products. ‐Field studies and bioaccumulation modelling to determine long‐term effects.
Toxicological Profiles	‐A lack of data about the chronic toxicity of nanoscale pigments in humans and animals. ‐Insufficient knowledge about synergistic effects between pigments and other environmental pollutants.	‐Long‐term exposure may cause undetected illnesses or ecosystem disruptions. ‐Combined effects can amplify toxicity or lead to novel hazards.	‐Toxicology studies with long‐term and low‐dose exposures. ‐Explore co‐exposure scenarios in controlled and field settings.
Health Impacts	‐Insufficient information about pigment‐induced microbial resistance in humans and animals. ‐Possible outcomes of pigments on endocrine and immune systems in animals and humans	‐Antimicrobial resistance (AMR) can be exacerbated, affecting global health. ‐The possibility of wide‐ranging health impacts, including hormonal disruption and weakened immunity, exists.	‐Monitor links to AMR in spots of pigment‐contaminants. ‐Detailed epidemiological and mechanistic studies.
Analytical Challenges	‐Detection of pigments in complex environmental samples is difficult at trace levels. ‐A lack of methods for distinguishing pigments from their transformation products in environmental and biological samples	‐Hidden hazards in the form of low‐level pigments and their decomposition products. ‐Underestimating Risks due to Misidentification	‐Developing more sensitive and selective analytical tools, such as mass spectrometry. ‐Use non‐targeted analysis in conjunction with machine learning to identify targets comprehensively.
Regulatory and Policy Gaps	‐Pigments are regulated differently in different countries and industries. ‐No lifecycle‐based risk assessment for pigments.	‐Poor management and control of pigment contamination worldwide. ‐Misses risks that arise in other lifecycle stages (production, use, disposal)	‐Align international guidelines and regulations in accordance with One Health approaches. ‐Use lifeLCA frameworks to assess risks holistically.
Social and Economic Dimensions	‐ Industries and consumers rarely know their pigment/health risks. ‐The economic costs of switching to safer, sustainable pigments remain unclear.	‐Contaminated products containing harmful pigments available widely without safety checks. ‐Adoption of green chemistry solutions is hindered by a lack of cost‐benefit analyses.	‐Industries and consumer targeted public awareness campaigns and educational programs. ‐Conduct cost/effectiveness studies for alternative green pigments.
Interdisciplinary Challenges	‐Lack of collaboration between environmental scientists, toxicologists, policymakers, and industry stakeholders.	‐ Delays the implementation of research on the ground.	‐Foster global interdisciplinary networks focused on pigment‐related risks.
Innovation Needs	‐Unexplored potential for green chemistry to redesign pigments for environmental safety. ‐No frameworks available to incorporate the One Health approach into pigmentation science.	‐The potential to substitute harmful pigments with more sustainable ones is still not explored. ‐Failure to consider the interconnectedness of risks in a systematic way.	‐Research and Development (R&D) on biogradable and non‐toxic pigments according to green chemistry principles. ‐Establish One Health‐based guidelines for pigment risk assessment and risk management.
Emerging Contaminants	‐Potential undeclared toxicity of engineered nanoscale pigments and their environmental persistence and transformation. ‐Inadequate awareness on the contribution of pigments to micro/nanoplastics spread.	‐Their tiny size and high reactivity may create unforeseen ecological and health threats. ‐Pigment‐containing plastics can increase pollution and ecosystem toxicity.	‐Include nanoscale pigment research in environmental monitoring programs. ‐Investigate pigment interactions with microplastics in both aquatic and terrestrial environments
Future Research Directions	‐No global pigment risk databases to use for research and policy. ‐Very few studies about how pigments are involved in climate change, their interaction with carbon cycles, etc.	‐Coordinated responses to pigment‐related risks are hindered. ‐Important processes like carbon storage in soils and aquatic ecosystems may be affected.	‐Develop a global open‐access database on the risks of pigments and ways to mitigate them. ‐Investigate effects of pigments on carbon cycling and climate models.

## 3 The One Health Framework

**Figure 2:**
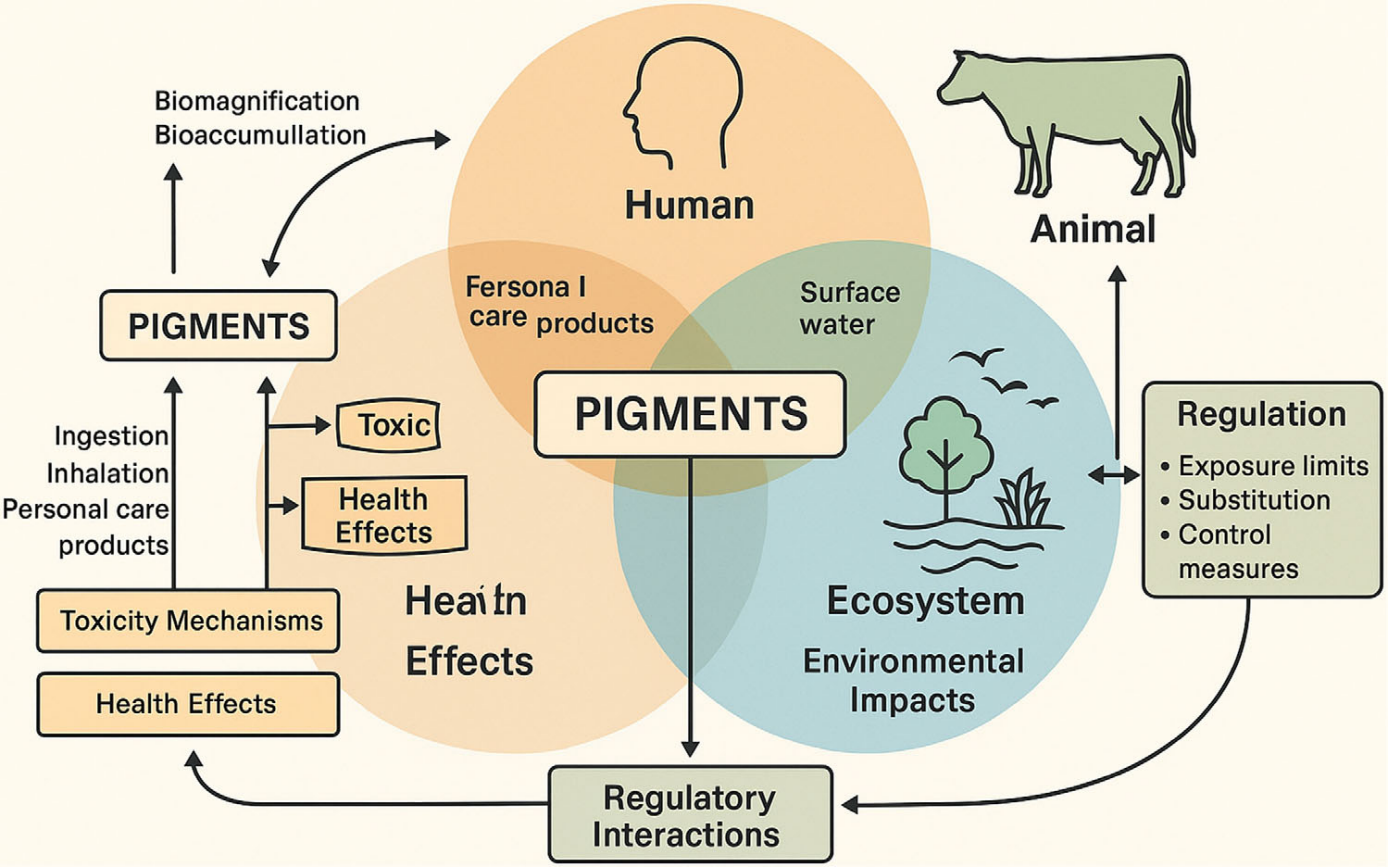
One Health Framework to assess Pigment risk. A detailed cross‐cutting schematic capturing the integrated ecological and health impacts of pigments within intertwined human, animal, and ecosystem domains. This framework illustrates complex bioaccumulation pathways, toxicity mechanisms, and regulatory interactions, shedding light on holistic risk characterization and sustainable environmental management (image drawn with Biorender and Canva.com).

A One Health approach recognizes that human, animal, and environmental health are intricately interconnected, as illustrated in **Figure** **2**. There is a growing demand for this framework to address complex challenges, including emerging contaminants, such as nanoscale particles like pigments, which could be multiple systems at once.^[^ ^11^ ^]^ The following sections discuss how a transdisciplinary approach can be used to effectively manage contaminants.

### 3.1 Conceptual Overview of One Health

One Health approach brings together knowledge from multiple sectors—medicine, veterinary medicine, environmental science, public health, etc. This comprehensive framing is appropriate because nanoscale pigments can create complex problems for the environment, biology, and the economy.^[^ ^12^, ^13^ ^]^ This article shows that ECs are the kinds of threats which require one Health approach to be managed because of their persistence, bioaccumulation tendencies, and complex interactions.^[^ ^14^ ^]^

### 3.2 Interdisciplinary Approaches to Nanoscale Pigments

Nanoscale pigments are a representative example of new chemical entities, which interact with biological systems and the environment in unique manners.^[^ ^15^ ^]^ The article (here) underlines the necessity of interdisciplinary research—across toxicology, materials science, ecology and policy studies—to understand the risks and benefits of these materials as a whole. This integration is best accomplished through the One Health framework, which promotes the sharing of methodologies and data across these areas.^[^ ^13^ ^]^

### 3.3 Impacts on Human, Animal, and Ecosystem Health

The entirety of the One Health triad as depicted in Figure 2 can be impacted by the production, use, and disposal of nanoscale pigments.

#### 3.3.1 Human Health

Nanoscale pigments can be inhaled, ingested, or absorbed through the skin, but their toxicological effects are largely unknown.^[^ ^13^ ^]^ The article raises concerns over bioaccumulation and toxicity, mirroring more general issues surrounding ECs.

#### 3.3.2 Animal Health

The pigments can bioaccumulate in wildlife and ecosystems, affecting their physiology and food webs. The research elaborates on the far‐reaching impact of ECs on ecosystems and offers valuable insights into the consequences of nanoscale pigments.^[^ ^3^, ^16^ ^]^

#### 3.3.3 Environmental Health

The usage of nanoscale pigments can pose the risk of environmental contamination during waste disposal, the degradation of consumer products, or through industrial processes. The study focuses on how ECs linger and change over time in the environment and the negative effects these changes can have on soil, water, and air quality.^[^ ^3^ ^]^

### 3.4 The Role of Equity and Inclusivity in One Health

The article notes the necessity of advancing social justice when dealing with the problem of emerging contaminants. People from marginalized demographic groups are adversely affected by pollution, and they cannot do anything to solve it. Such gaps are sought to be filled with the help of inclusive policymaking and fair technological and scientific dissemination through the One Health approach to policy.^[^ ^17^ ^]^

### 3.5 Urgency of Action and Global Collaboration

Emerging contaminants like nanoscale pigments are a global problem that needs international cooperation. With the One health framework, such integration becomes possible as there is an avenue to design policies and solutions that are sustainable. To reduce the disadvantages that come with nanoscale pigments, an integrated approach between governments, industries, and academia is necessary.^[^ ^18^ ^]^ This perspective on nanoscale pigments, based on the One Health framework, provides insight into why others should consider the implications of nanoscale pigments more comprehensively. The following sets up the stage in which these properties, pathways, and impacts will be looked at in the next sections.

## 4 Properties of Nanoscale Pigments

The large surface area and small size of nanoscale pigments has modified their electronic and optical properties, making them readily distinguishable physicochemically. Such characteristics allow these pigments to work well in many applications and at the same time affect their bioavailability as well as the environment.^[^ ^19^ ^]^ These properties must be considered due to their relevance to the broader issue of emerging contaminants.

### 4.1 Nanostructure‐Dependent Optical and Chemical Properties

The nanoscale sizes of these pigments give them unique optical characteristics such as improved color vibrancy, stability, and tunability that make them important in coatings, cosmetics, and biomedical imaging.^[^ ^20^ ^]^ These properties arise from quantum effects and size‐dependent light–matter interactions, like scattering and absorption.^[^ ^21^ ^]^ The chemical reactivity of nanoscale pigments can be beneficial for catalysis and material development, but it poses a health and environmental risk due to potential biological reactivity.^[^ ^22^ ^]^

### 4.2 Surface Area and Functionalization

**Figure 3:**
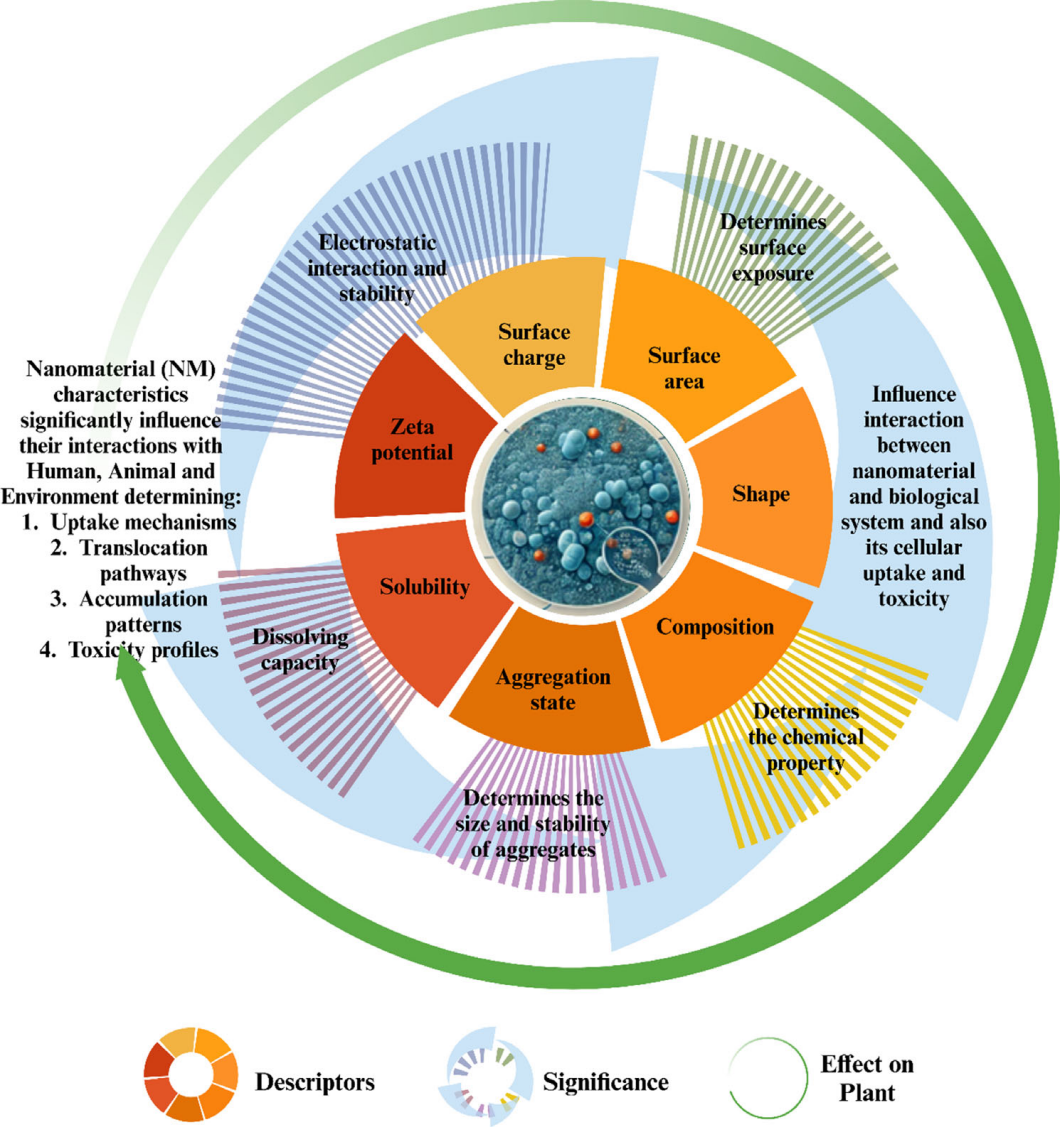
Schematic representation of nanoscale pigments highlighting their structure‐dependent properties—optical, chemical, and thermal stability. The illustration shows their interactions with environmental matrices (water, soil), as well as molecular‐level transformations affecting bioavailability^[^ ^92^ ^]^ (image drawn with Biorender.com).

Due to their nanoscale size, the surface area‐to‐volume ratio of pigment particles is much higher, which means they can interact more with the surrounding media as shown schematically in **Figure** **3**. This chemical uniformity benefits their application, but also makes them more likely to adsorb other pollutants, and changes their behavior in biological and environmental systems.^[^ ^23^ ^]^ Surface functionalization, widely used to enhance stability or compatibility for pigments, additionally affects their interactions with living organisms and ecosystems.^[^ ^24^ ^]^

### 4.3 Environmental Transformations and Persistence

From nanomaterials to polymers, ECs in the environment are changing complex environmental processes. Natural organic matter, ions, and microorganisms at the nanoscale can interact with pigments and change their chemical structure and aggregate state. These transformations impact their persistence, mobility, and bioavailability.^[^ ^25^ ^]^ For example, pigments could accumulate in aquatic environments,^[^ ^26^, ^27^, ^28^ ^]^ limiting their spread and enhancing local ecological effects.^[^ ^29^, ^30^ ^]^

## 5 Environmental Pathways and Fate of Nanoscale Pigments

As ECs, nanoscale pigments can be emitted into the environment through various routes, including industrial effluent, product degradation, and disposal. Emergent pollutants each have their own physicochemical properties, which invariably have implications for their fate, transport, and transformation throughout ecosystems.^[^ ^31^ ^]^

### 5.1 Sources of Environmental Pigment Contamination

Nanoscale pigments can be released into the ecosystem through many processes, which is associated with negative effects on ecosystems, humans, and sustainable development. Factories emitting during production of factorymade goods is a critical source, as pigments are released into the atmosphere and water bodies, often evading traditional waste management systems.^[^ ^4^ ^]^ At the same time, the use and degradation of consumer products (coatings, textiles, plastics, etc.) lead to the progressive loss of pigments as these products age. These pigments are released into the terrestrial and aquatic environments, leading to the introduction of nanoparticles to the environment.^[^ ^32^ ^]^

Furthermore, disposal of pigment‐carrying products in landfills contributes to environmental contamination, as landfill gases and leachates promote the leaching of pigments to surrounding soils and water bodies, leading to the ongoing pollution of surrounding terrestrial and aquatic systems. This release mechanism suggests the interconnectedness of industrial practices, waste management, and ecological health.^[^ ^33^ ^]^ A holistic One Health approach, integrating scientific innovation, policy frameworks, and sustainable practices, is needed to mitigate the environmental and health impacts of nanoscale pigments.

### 5.2 Mechanisms of Transport in Ecosystems

**Figure 4:**
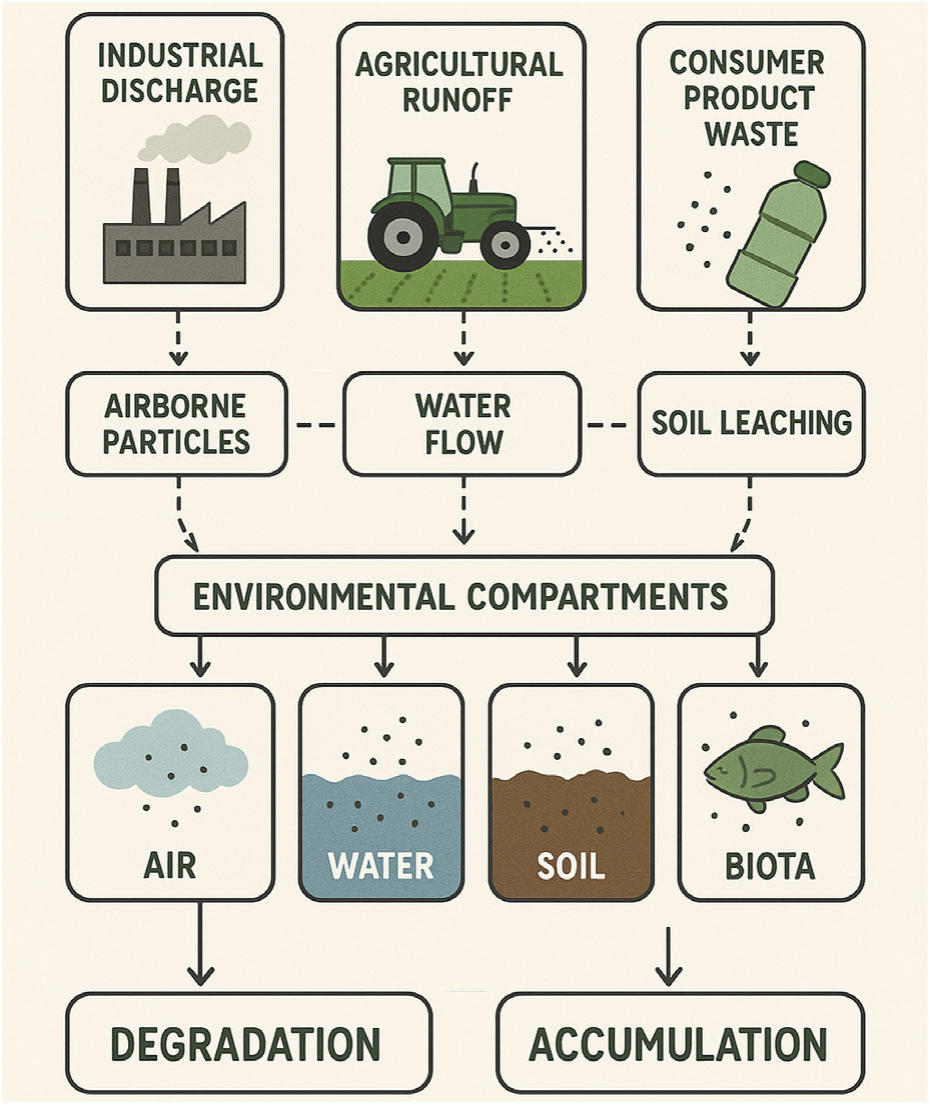
Environmental Pathways of Nanoscale Pigments. The flowchart shows the environmental fate of nanoscale pigments. It starts with industrial discharge, agricultural runoff, and consumer product waste. Transport mechanisms, such as air particles, water runoff, and soil leaching, are shown in the next arrows in the diagram. Environmental compartments (air, water, soil, and biota) are displayed as well transformation processes such as degradation, agglomeration, and surface change. The scheme highlights fate and behavior aspects, environmental impacts, and challenges in monitoring and risk assessment. It concludes with potential mitigation strategies, providing a comprehensive overview of the complex interactions between nanoscale pigments and the environment (image drawn with Biorender and DALL‐E3).

Nanoscale pigments possess different transport and fate mechanisms that are also influenced by their interactions with environmental media, size, and surface charge properties after their release into the environment, as demonstrated in **Figure** **4**. Alternatively, dissolution and dispersion in aquatic pathways depend on pH, ionic concentration, and the presence of organic compounds, as these influence pigment mobility or biological availability in water systems.^[^ ^34^ ^]^ Pigments that remain immobile but active on soil and sediment particles for years can change soil composition, affecting its health, and even leach to contaminate ground water. Airborne transport are also important as electron‐dense nanoscale pigments or their aggregates can be dispersed at high altitudes and result in long‐range deposition in distant ecosystems.^[^ ^35^ ^]^ It is clear that nanoscale pigments are transported across air, water, and soil systems, underscoring the need to better understand their intersystemic transfer and ecological impact.

### 5.3 Transformations in Environmental Compartments

The behavior, persistence, and ecological impacts of nanoscale pigments are largely determined by the physical, chemical, and biological transformations that they undergo. In aquatic systems, such changes in the distribution of pigments resulting from physical processes during degradation (e.g., aggregation and sedimentation) may enrich sediments with pigments and thus influence benthic ecosystems. Different chemical transformations, including oxidation, reduction, and reactions with natural organic matter, can change the structural and reactive properties of pigments, affecting their toxicity, mobility, and bioavailability. These transformations reveal the importance of considering the behaviors of nanoscale pigments in the environment when assessing their potential risks to the environment and health. Understanding these processes is essential for risk assessment frameworks and guidance for sustainable management strategies leveraging the One Health paradigm that supports the safe use of nanoscale pigments, while protecting environmental and human health.^[^ ^36^ ^]^

### 5.4 Bioaccumulation and Trophic Transfer

Nanoscale pigments are a novel class of ecotoxicants with potential for bioaccumulation and biomagnification through food webs that threaten ecosystems and human health. In water, pigments may be taken up by primary producers (like algae or zooplankton) and biomagnify as they are transferred to successive higher trophic levels (fish and predators).^[^ ^3^ ^]^ In terrestrial ecosystems, adsorption on plant roots or consumption by the soil fauna transfers pigments into terrestrial food webs and increases exposure of a diversity of organisms to toxicity.^[^ ^37^ ^]^ Humans are primarily exposed through contaminated water or food, resulting in at‐risk populations. Thesebioaccumulation dynamics of nanoscale pigments are similar to those for other engineered contaminants, raising important questions about their long term effect on ecosystem and human health In order to mitigate risks and align with the One Health approach, which integrates environmental, animal, and human health considerations; the findings underscore the urgent need for preventive measures, such as enhanced monitoring, regulatory frameworks, and sustainable nanomaterial design.^[^ ^38^ ^]^

### 5.5 Knowledge Gaps and Future Directions

While our knowledge of many engineered contaminants is making progress, relevant knowledge on nanoscale pigments is still lagging behind, hampering adequate risk assessment and sustainable management solutions in the field. Although these pigments have high specificity to their sources, complex environmental matrices continue to hinder accurate detection and quantification, calling for advanced chemianalytical techniques for enhanced precision and reliability.^[^ ^39^ ^]^ Long‐term ecological impact studies would be equally important for better understanding of their impacts on biodiversity and ecosystem. One way to address these gaps is to have an interdisciplinary research agenda that integrates innovative methodologies and promotes collaboration across scientific, policy, and sustainability domains.^[^ ^13^ ^]^ This section explores nanoscale pigment's environmental pathways and fates in order to understand their potential risks and formulate strategy for managing them sustainably.

## 6 Health Implications of Nanoscale Pigments

### 6.1 Direct Human Health Impacts

Through various exposure routes, including inhalation, ingestion, and dermal contact, nanoscale pigments pose significant health risks to humans.^[^ ^40^ ^]^ For instance, the inhalation of airborne nanoscale pigments can contribute to respiratory inflammation, oxidative stress, and chronic diseases like fibrosis or asthma, especially in occupational contexts or polluted environments.^[^ ^41^ ^]^ Dermal and mucosal exposures (common of cosmetics or coatings) is where pigments pass the skin barrier layers or mucosal layers and can build localized irritation or systemic toxicity.^[^ ^42^ ^]^ Oral ingestion through contaminated crops or water is another major route that incorporates pigments into the gastrointestinal tract, which in turn can be the cause of systemic illnesses. This is not just a concern specific to nanoscale pigments (or pigments in general), but also other engineered contaminants, mostly nanoparticles, which also possess the properties of crossing biological barriers and raising accumulation in sensitive biological tissues. These exposure pathways and their potential health effects must be understood so that preventive strategies and regulatory priorities can be designed to address these in the context of One Health, minimizing risk to human health alongside environmental and ecological balance.^[^ ^43^ ^]^

### 6.2 Animal Health and Ecosystem Impacts

Animal health is significantly impacted by the direct and indirect infiltration of these nanoscale pigments into ecological systems, which will ultimately have an impact on ecosystem stability and biodiversity. Pigment exposure through contaminated water or sediments can also impair reproduction, development, and survival in aquatic organisms and other wildlife.^[^ ^44^ ^]^ These pigments enter the food chain where trophic magnification results in higher concentrations of these compounds in the predators because they prey upon contaminated sources and this ultimately increases exposure to these compounds.^[^ ^45^, ^46^ ^]^

### 6.3 Mechanisms of Toxicity

The health effects of the nanopigment are based on their mechanisms of toxicity which could be harmful to human beings as well as for the animals. One crucial metabolic action is the production of oxidative stress, where properties of reactive nanoscale pigments give rise to reactive oxygen species and cause cellular damage, inflammation, and tissue injury.^[^ ^47^ ^]^ Furthermore, certain class of these pigments can be genotoxic and/or carcinogenic via interaction with DNA or through mutation or activation of cancer pathways, making health effects of long‐term exposure a serious concern^[^ ^48^ ^]^ In addition, these nanoscale pigments have the potential to interfere with immune response, thus, causing hypersensitive reactions or immunosuppression which weakens the body from combating infections and diseases.^[^ ^49^ ^]^ These toxicological mechanisms characterize the wider array of engineered contaminants, as formerly reported.^[^ ^50^ ^]^

### 6.4 Addressing Health Risks within the One Health Framework

The interrelated effects of nanoscale pigments on human, animal, and environmental health highlight the need for a One Health approach, focused on the integration of preventive measures,^[^ ^51^, ^52^ ^]^ policy mechanisms, and collaborative research. Green chemistry principles should be incorporated into the R&D strategies in order to make green pigments with the least ecological footprint possible to minimize the harm to the ecosystem.^[^ ^53^ ^]^ At the same time, robust policy frameworks must be established at international levels to regulate the production, use, and disposal of nanoscale pigments, ensuring that exposure risks are minimized at every stage. Collaborations between different disciplines are equally important for identifying common risks and developing crossdisciplinary solutions. The monolithic solutions crafted to tackle these diverse issues must also establish an interconnected exchange to discuss, rethink, and create pathways for the exploration of engineered contaminants—nanoscale pigments.

### 6.5 Recommendations for Health‐Centric Approaches

A holistic approach is needed to mitigate the health and environmental hazards posed by nanoscale pigments. Enhanced monitoring should develop detection tools to accurately assess pigment exposure in different environmental settings, leading to improved risk assessments.^[^ ^54^ ^]^ Quantitative mechanistic models of pigment toxicity need to be developed, providing a data‐driven basis for regulatory decisions.^[^ ^55^ ^]^ Public awareness campaigns are also important for educating stakeholders about the safe use and disposal of pigment containing products and promoting responsible consumer practices. These recommendations are consistent with the article's emphasis on proactive contamination control and sustainable strategies.

## 7 Innovative Applications and Sustainable Practices

Nanoscale pigments are a new class of materials coming from an area of emergent materials science that can have use and impact across various industries. The high color intensity stability and multifunctionality of properties has enabled technological advances in recent applications in health and sustainable sectors.^[^ ^56^ ^]^ However, the study highlights the need to make sustainability and green chemistry principles part of their lifecycle in order to reduce environmental and health risks.^[^ ^57^ ^]^

### 7.1 Advanced Applications of Nanoscale Pigments

Nanoscale pigments are extremely versatile and thus are critical for numerous advanced technologies. Thereby optimizing the light absorption and emission response, photonic and optoelectronic devices take advantage of this property for sensors, display technologies, and photovoltaic cells.^[^ ^55^ ^]^ Nanopigments have several advantages in biomedical applications such as drug delivery, imaging, and diagnostics owing to their ability to fine tune surface properties and the presence of a high level of biocompatibility, but more refinement is likely needed for their optimisation.^[^ ^58^ ^]^ These pigments are beneficial in functional coatings and provide self‐cleaning, antimicrobial, and UV‐resistant properties, thus increasing durability and functionality in coatings.^[^ ^59^ ^]^ In textiles and cosmetics, nanoscale pigments enhance durability, brightness, and resistance to environmental degradation. These examples illustrate the larger trend of seeking to harness the capabilities of nanoscale materials for new technology, while also underlining the need for careful consideration of the risks involved.^[^ ^60^ ^]^

### 7.2 Role of Green Chemistry in Nanoscale Pigment Design

**Figure 5:**
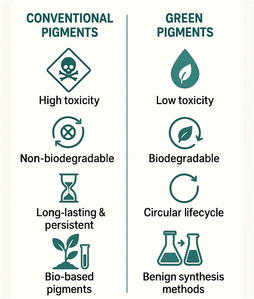
Green Chemistry Principles in Sustainable Pigment Design. This comparative diagram illustrates the paradigm shift from conventional pigment production to sustainable pigment innovations guided by green chemistry principles. The figure highlights key differences in toxicity profiles, biodegradability characteristics, and LCAs between traditional and ecofriendly pigment design approaches.

Integrating green chemistry principles into the production of nanoscale pigments is essential to mitigate their environmental and health impacts while advancing sustainable material design as proposed in **Figure** **5**. A “Benign‐by‐and‐for‐Design” approach can be employed to tailor pigments with reduced toxicity and enhanced biodegradability, ensuring they are safer for ecosystems and human health.^[^ ^61^ ^]^ Furthermore, reducing the ecological footprint of pigment manufacturing requires optimizing raw materials and the energy needed to produce them. Safeguarding the global environment will also require the development and use of safer alternatives, removal of toxic heavy metals/hazardous substances, etc. The same concepts discussed in this context are also being explored more broadly in the realm of engineered contaminants for the design of safer, more sustainable materials. This perspective argues that sustainability is not only a solution to the challenges posed by nanoscale pigments but also a proactive approach to addressing global environmental and health concerns.^[^ ^62^ ^]^

### 7.3 Circular Economy and Lifecycle Management

Nanoscale pigments can be made sustainable through a circular economy model that considers the entire life cycle from manufacture to application to disposal, which is good for humans and the environment.^[^ ^57^ ^]^ This method is critical to reducing waste, as upcycling pigments from end‐of‐life products conserves raw materials while reducing environmental destruction. Also, ecodecomposition is essential because they allow for the engagement of the pigments to ensure their disposal, which prevents contamination of ecosystems and maintains the quality of air, water, and soil. Lifecycle assessment (LCA) tools should be used to fully understand the environmental impact of nanoscale pigments, analyzing their impacts from production to end‐of‐life stages.^[^ ^32^ ^]^ This article emphasizes how lifecycle approaches to emerging contaminants management can mitigate negative health and environmental effects of nanoscale pigments.

### 7.4 Innovations in Remediation and Restoration

Although nanoscale pigments are frequently mentioned in terms of the environmental hazards they pose and their toxicity, they can also be applied to environmental restoration and remediation, thus presenting a potential breakthrough in the field of environmental protection. Active pigments‐based water purification: Active pigments can be engineered to adsorb or degrade contaminants, offering efficient and scalable strategies for the treatment of aqueous sources in which impurities should be removed.^[^ ^63^ ^]^ The same applies for pigments with catalytic or absorbent properties, which can be used to improve detoxification of polluted soils in soil remediation processes and ecological functional restoration. Coatings with nanoscale pigments can also be engineered to degrade airborne contaminants and thus could play an important role in enhancing air quality in urban and industrial environments. Such applications demonstrate that engineered contaminants can also be leveraged for sustainable innovation.^[^ ^64^ ^]^ Taking advantage of their unique properties responsibly can help address global environmental issues while aligning with the principles of One Health, ensuring ecological restoration and human well‐being.

### 7.5 Policy and Industry Standards for Sustainable Practices

An in‐depth understanding of nanoscale pigments may cast light on the need for making solid policies and industrial standards for their effective utilization. Ensuring uniform standards that are considered safe and environmentally beneficial across international borders also includes prepared statutes, such as global regulations regarding nanoscale pigments, influencing their production, use, and disposal.^[^ ^65^ ^]^ The internal regulation of business practices should also be encouraged, which would drive producers to utilize best practices and openly communicate pigment composition ingredients for greater accountability and consumer trust. Furthermore, cross‐sector collaboration will be vital, ensuring that governments, industries, and academia work hand in hand in fostering and applying concrete tax to safeguard against the potential perils of nanoscale pigments.^[^ ^66^ ^]^

### 7.6 Opportunities for Future Research

As nanoscale pigments become a key focus of innovation, an emphasis is being placed on developing pigments of the future that are both high performing and environmentally friendly. This needs tests including field studies—to assess the long‐term behavior of the pigments in different environmental settings to understand the impact across ecosystems. Finally, interdisciplinary approaches are warranted to address critical knowledge gaps, including those in material science, toxicology, and environmental science, to develop a more comprehensive understanding of the full scope of the biological interactions impacting nanoscale pigments. Using nanoscale pigments as an example, this article argues that a more future‐oriented approach to research is needed to create effective management opportunities for emerging contaminants. It is possible to use these materials in new ways that promote environmental and technical advancements, thus aligning the concept of One Health with innovation. It is through this approach that nanoscale pigments are viewed from an equitable and global perspective, ensuring the sustainability of both the human and ecological systems.^[^ ^67^ ^]^

## 8 Detection and Analytical Advances

Nanoscale pigments have special characteristics (i.e., small particle size, surface reactivity, and complex interactions with environmental and biological systems) that require sophisticated analytical tools in order to detect and characterize them as discussed in the following sections.

### 8.1 Advances in Analytical Techniques and their suitability

The detection and characterization of nanoscale pigments have significantly advanced with recent developments in analytical chemistry, enabling a more detailed and accurate understanding of these materials and their behavior in the environment. High‐resolution mass spectrometry (HRMS) is a powerful tool that allows for the identification of pigments and their transformation products with a high level of specificity, even when present at trace levels, making it invaluable for detecting subtle environmental impacts.^[^ ^68^ ^]^ Nuclear magnetic resonance (NMR) spectroscopy provides detailed structural information that is essential for understanding how pigments interact and undergo transformations in various environments, offering insights into their stability and reactivity.^[^ ^69^ ^]^ Additionally, electron microscopy, including techniques like transmission electron microscopy and scanning electron microscopy, allows for the direct visualization of nanoscale pigments and their agglomerates, providing critical information on their morphology and dispersion. These innovative approaches enhance the discovery approaches covered in the article, which highlights the significance of reliable and adaptable techniques for practical analysis of emerging contaminants.^[^ ^70^ ^]^

### 8.2 Challenges in Detecting Nanoscale Pigments

The low concentrations, complex mixtures, and dynamic behavior of nanoscale pigments in environmental and biological systems pose significant analytical challenges. Moreover, pigments undergo dynamic environmental interactions with diverse phases including agglomeration, solubilization, and alteration, which can complicate their identification and quantification as these processes may conceal their real identity and distribution.^[^ ^71^ ^]^ Also, nanoscale pigments are seldom present in pure form; they generally occur in complex mixtures necessitating sophisticated analytical approaches with the potential to deconvolute these complex matrices. Additionally, their generally low environmental concentrations make their accurate measurement via conventional detection systems, such as high‐performance liquid chromatography (also called high‐pressure liquid chromatography), very challenging in the absence of interference from co‐occurring constituents due to poor sensitivity or selectivity. These challenges are not exclusive to nanoscale pigments, but they are also relevant issues for other engineered contaminants, as noted earlier. These difficulties will challenge the future generation of creative, intense, and selective detection procedures, to allow researchers to characterize the environmental fate, behavior, and effects of the nanoscale pigments.^[^ ^72^ ^]^

### 8.3 Emerging Bioanalytical Tools and New Approach Methods (NAMs)

By combining AI & ML, new opportunities for assessing the biological interactions of nanoscale pigments are emerging, offering effective platforms for detecting and understanding their effects.^[^ ^73^ ^]^ As a result of the development of novel biosensors, particularly aptamer‐based biosensors, pigments can be detected quickly and precisely in complex biological and environmental matrices, facilitating real‐time monitoring.^[^ ^74^ ^]^ Moreover, effect‐directed analysis combines chemical analyses with bioassays to identify pigments that display significant biological activity, thereby enabling the identification of those that can pose risks to ecosystems (e.g., algae) or human health.^[^ ^75^ ^]^ In addition, quantitative polymerase chain reaction (qPCR) and next‐generation sequencing, as genomic tools, can also detect biological effects, such as changes in gene expression induced by exposure to nanoscale pigments. These bioanalytical innovations are crucial to understanding emerging contaminants such as nanoscale pigments, with a focus on integrating these techniques.^[^ ^76^ ^]^

### 8.4 Nontargeted and Suspect Screening Approaches

In order to detect and classify nanoscale pigments and to identify unknown pigment composition, nontargeted screening and suspect screening have emerged as effective strategies. The mass spectra are analyzed without prior knowledge of the specific compounds in the samples, allowing for the potential to discover unknown pigments that were previously unrecognized.^[^ ^77^ ^]^ Instead, suspect screening employs extensive chemical libraries and databases for targeted searches of pigments and their derivatives, making it easier to efficiently identify known and potentially harmful pigments. Additionally, the combination of spectral information with machine learning‐based predictive models has allowed the identification and classification of pigments to be greatly improved utilizing spectral features to make more precise determinations.^[^ ^73^ ^]^ Advanced screening approaches, such as nanoscale pigments, are essential for revealing the full spectrum of emerging contaminants, including their presence and effects in diverse environments, as outlined in the article.

### 8.5 Real‐Time Monitoring Technologies

In order to collect data efficiently and continuously, real‐time monitoring systems are crucial for tracking nanoscale pigments in the environment and biological systems.^[^ ^73^ ^]^ It is possible to detect pigments on‐site in air, water, and soil with portable mass spectrometers equipped with molecular compositions and mass spectral characteristics of pigments from different groups.^[^ ^78^ ^]^ In particular, optical sensors can measure the absorption, reflection, or emission spectra associated with each pigment, which can offer a rapid and nondestructive method to characterize environmental samples and supply qualitative or quantitative information regarding pigments in real time.^[^ ^79^ ^]^ Comprising a network of several sensors, integrated IoT systems are also growing in scope to cover more general environmental exposure areas and are coupled with data analytics systems for continuous, automated monitoring. Emerging pollutants like nanoscale pigments depend on these real‐time monitoring technologies to be managed and to guarantee a quick reaction to environmental hazards. As the work emphasizes, the integration of such systems is essential to raise our capacity to monitor and control the influence of nanoscale pigments and other ECs in the surroundings.^[^ ^80^ ^]^

### 8.6 Gaps and Future Directions

**Figure 6:**
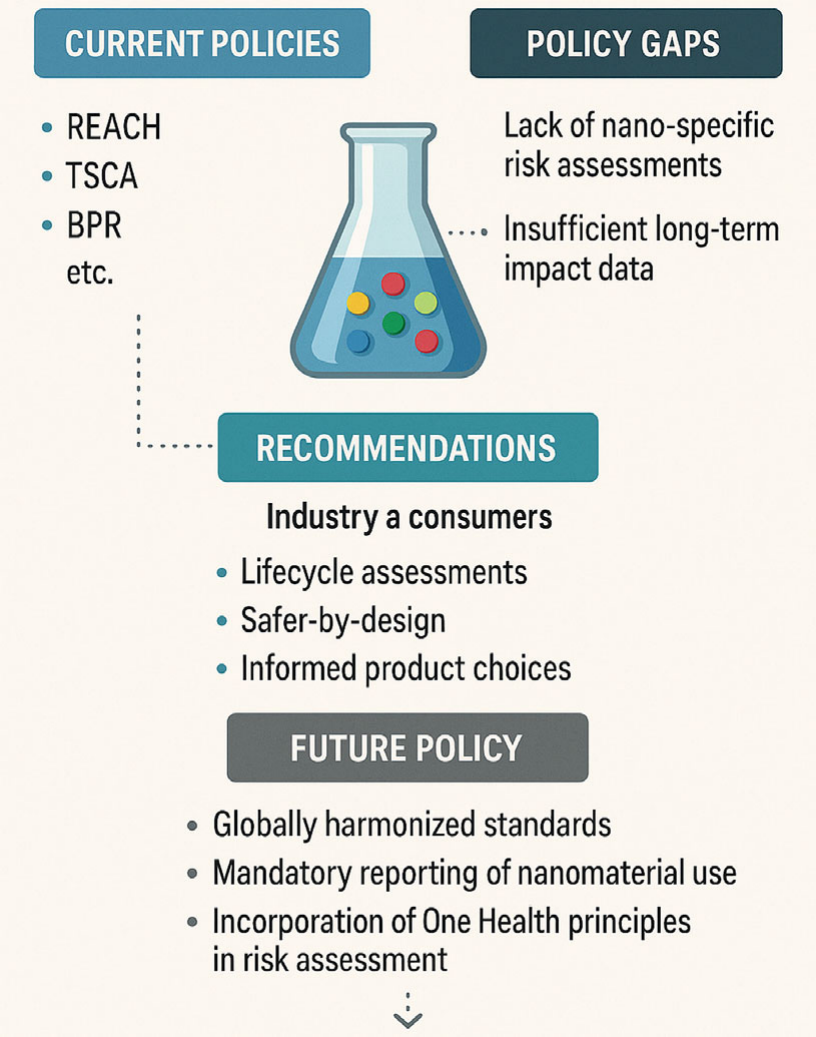
Policy Gaps and Regulatory Recommendations for Nanoscale Pigments. This comparative infographic illustrates the current regulatory landscape for nanoscale pigments, highlighting existing policies (e.g., REACH, TSCA, BPR) alongside identified gaps in regulation. The figure outlines major policy gaps, including the absence of a standardized nanospecific risk assessment framework and a lack of data regarding long‐term impacts. The recommendations focus on LCAs, safe‐by‐design practices, and informed product selections for consumers and industry.^[^ ^3^ ^]^ We outline key recommendations to guide future policy, such as establishing globally harmonized standards, mandatory reporting of nanomaterial deployment, and the application of a One Health approach to risk assessment. Such cross‐national comparison highlights the importance of the adoption of a more coordinated, global approach to nanoscale pigment regulation to promote environmental sustainability and the protection of public health (image drawn with Biorender and DALL‐E3).

As demonstrated in **Figure** **6**, despite advancements in analytical techniques, significant gaps remain in the detection and analysis of nanoscale pigments, hindering comprehensive risk assessment and management. The lack of standardized protocols for sampling, preparation, and analysis creates inconsistencies in data, underscoring the need for harmonized methodologies to ensure reproducibility and comparability across studies.

Furthermore, identification of secondary transformation products of pigments in biological and environmental systems is difficult since these byproducts might show different toxicological profiles and ecological effects. Bridging these gaps calls for multidisciplinary cooperation combining analytical chemistry with toxicology and ecology to offer a whole knowledge of pigment behavior and effects.^[^ ^49^ ^]^ In order to study engineered contaminants effectively, we advocate for focused research and investment in developing innovative tools and approaches. Scientists can better describe the behavior, hazards, and environmental fate of nanoscale pigments using advanced detection technologies and supporting multidisciplinary collaborations. Dealing with the problems presented by new materials, pushing forward knowledge, and guiding sustainable policies inside the One Health framework depend on this cooperative effort.

## 9 Policy and Regulatory Perspectives

On the road toward acceptive use, there must be strong regulations—as to how to safely quantify nanoscale pigments as emerging contaminants. Reflecting from the regulatory lens, this report is important as it calls to think global and across disciplines to tackle the complex nature of the challenges posed by ECs and offers some key ecommendations toward shaping policies on nanoscale pigments.

### 9.1 Current Regulatory Landscape

The currently fragmented regulatory framework for nanoscale materials, including pigments, lacks specificity, and fails to ensure safety. International guidelines, including OECD and ISO nanomaterials safety documents, have a wide‐ranging scope but do not consider the specific properties and likely risks of the nanoscale pigments.^[^ ^81^ ^]^ However, at the national level, regulatory moves in the USA (through the EPA) and the EU (under REACH) have initiated coverage related to nanomaterials but still suffer from substantial coverage gaps regarding pigments.^[^ ^82^ ^]^ While guidelines can instill confidence in safety protocols, these voluntary industry best practices are nonbinding by nature and vary from market to market.^[^ ^83^ ^]^ To overcome these weaknesses, a concerted approach is needed in the form of established harmonized and pigment‐specific regulations as well as international cooperation and, in some cases, enforceable industry standards. These efforts will ensure that nanoscalepigments can be produced, used, and disposed of safely, considering all relevant health endpoints in the One Health framework and supporting innovative sustainability solutions.

### 9.2 Challenges in Regulating Nanoscale Pigments

Effective regulation of nanoscale pigments is lagging behind due to some major challenges in comprehensive risk management and policy development. The broad diversity of nanoscale properties, such as size, surface chemistry, and degree of functionalization, impacts toxicity, environmental persistence, and bioaccumulation, posing a challenge to risk assessment.^[^ ^83^ ^]^ Furthermore, a lack of health and environmental impact data hinders the development of evidence‐based policy and safety guidelines. Additionally, the worldwide aspects of pigment trade and dissemination contribute transboundary footprints that require global coordination and harmonized regulatory strategies to address. A number of these roadblocks are indicative of broader challenges in governing emerging contaminants, which are highlighted in the article, and highlight the importance of adopting precautionary measures and fostering international collaboration for EC governance to be effective.^[^ ^54^ ^]^ With an emphasis on pigments, **Table** **2** provides an overview of the current regulatory environment surrounding nanomaterials. It highlights the disparities in specificity, strengths, and enduring gaps among major frameworks like the US TSCA, EU REACH, and OECD guidelines. In order to better align innovation with safety and sustainability imperatives, the article makes recommendations for improving regulatory coherence. These recommendations include mandatory nanospecific reporting for pigments, LCA integration, and encouraging benign‐by‐design approaches.^[^ ^84^, ^85^, ^86^, ^87^, ^88^, ^89^, ^90^ ^]^

**Table 2:** Comparative regulatory approaches for nanomaterials (with pigment relevance).

Regulatory Body/Framework	Primary Scope	Key Provisions for Nanomaterials	Specificity for Nanoscale Pigments	Strengths	Weaknesses/Gaps	Proposed Enhancements (from paper's recommendations)
EU REACH	General chemicals	Explicit nano‐specific requirements for registration (since Jan 2020).^[^ ^75^ ^]^ Nanoforms assessed separately if different risks.^[^ ^9^ ^]^ Updated IUCLID tools, ECHA guidance.^[^ ^75^ ^]^	Explicit requirements apply to nanoforms of substances, including pigments. EU Cosmetics Regulation specifically bans/restricts certain nano‐pigments.^[^ ^76^ ^]^	Comprehensive, precautionary, industry responsibility for data.^[^ ^77^ ^]^ “Right to know” for consumers.^[^ ^77^ ^]^ Progress towards explicit nano‐specific provisions.^[^ ^75^ ^]^	Initial lack of specific nano‐provisions.^[^ ^77^ ^]^ “Phase‐in substance” loophole for pre‐2008 NMs.^[^ ^78^ ^]^ Lack of specific worker protection regulations for NMs.^[^ ^75^ ^]^ Implementation challenges for diverse nanoforms.	Mandatory nano‐specific reporting for all pigments. Integration of LCA. Incentivizing benign‐by‐design. Targeted bans for high‐risk pigments. Enhanced international collaboration.
US TSCA	Chemical substances	Nanoscale materials are “chemical substances”.^[^ ^79^ ^]^ New NMs require EPA review (PMN). One‐time reporting for existing NMs regardless of volume (2017 rule).	Pigments are covered as “chemical substances” under TSCA. New nano‐pigments require PMN. Existing nano‐pigments subject to one‐time reporting.	Strong framework for new chemical review.^[^ ^79^ ^]^ Nanoscale Materials Stewardship Program (NMSP) for voluntary data.^[^ ^12^ ^]^	“Unreasonable risk” threshold can be reactive. Voluntary programs may lack comprehensive data. Uncertainties in identification/characterization of new nano‐properties. Less explicit mandates than evolving REACH.	Mandatory nano‐specific reporting for all pigments. Accelerated harmonization of international standards. Integration of LCA. Incentivizing benign‐by‐design. Targeted bans for high‐risk pigments. Enhanced cross‐sectoral collaboration.
OECD Guidelines	Nanomaterials, advanced materials	Promotes international cooperation on safety.^[^ ^80^ ^]^ Develops standardized test methods (TGs) and Guidance Documents (GDs) for characterization, fate, effects.^[^ ^80^ ^]^ Focus on bridging innovation‐risk assessment gap.^[^ ^80^ ^]^	Applicable to all manufactured nanomaterials, including pigments, for standardized testing and characterization.	Crucial for global consistency in data generation and comparability.^[^ ^4^ ^]^ Addresses fundamental data needs for risk assessment.	Guidelines are non‐binding, relying on voluntary adoption. Slow development of new test methods.^[^ ^49^ ^]^	Accelerated harmonization of international standards. Mandatory nano‐specific reporting. Integration of LCA. Incentivizing benign‐by‐design. Enhanced cross‐sectoral collaboration.

### 9.3 Risk Assessment Frameworks

There is need to set up a more effective risk assessment framework for nanoscale pigments, it is crucial to consider the unique properties of these pigments and their interactions with the environment, which can be achieved through a more flexible and comprehensive strategy. Pigments, from production to disposal, have health and environmental impacts, making lifecycle analysis critical for impact evaluation. This global aspect also helps understand possible hazards during the whole lifecycle of the pigment.^[^ ^91^ ^]^ A tiered risk assessment approach should include both quantitative and qualitative assessments to ensure that regions with little data are included as well, which can strengthen risk assessments. Policies that enable regulations to adapt to scientific advances are also essential, ensuring that safety standards remain up to date with the times. An adaptive and proactive risk characterization framework is crucial for the management of emerging contaminants, and this iterative management approach aligns directly with those recommendations.^[^ ^92^, ^93^, ^94^, ^95^, ^96^, ^97^, ^98^, ^99^, ^100^, ^101^ ^]^

### 9.4 Actionable Policy Recommendations

The section now offers concrete, *actionable policy recommendations* specifically tailored for nanoscale pigments under the One Health umbrella, grounded in the preceding critical analysis. These include the following. 1) Mandatory nanospecific reporting and data generation. 2) Accelerated harmonization of international standards. 3) Incentivizing “Benign‐by‐Design” and circular economy models through concrete policy mechanisms. 4) Integration of LCA into regulation. 5) Targeted restrictions/bans for high‐risk applications, drawing lessons from precedents like the EU Cosmetics Regulation. 6) Enhanced cross‐sectoral and international collaboration. 7) Extending the “Right to Know” for nanoscale pigments in consumer products.

## 10 Conclusion

The rigorous application of the One Health lens in this article provides a comprehensive and forward‐looking perspective on nanoscale pigments. The manuscript effectively highlights the dual nature of pigments as both innovation enablers and emerging contaminants, offering a practical roadmap for balancing these aspects.^[^ ^102^ ^]^ There are numerous pathways in which these pigments can interact, including bioaccumulation in food webs, transformation in the environment, or they can persist in ecosystems. Nanoscale pigments have been applied in numerous settings; however, important knowledge gaps persist concerning nanoscale pigment behaviors, their toxicological implications, and their ecological consequences in heterogeneous environmental contexts. Filling these knowledge gaps is imperative so that risk assessments and management are tailored to risk. Incorporating One health approach affirms the interconnectedness between human health and health of animals and environment which is necessary for informative assessment of risks associated with pigments. It is possible to safeguard the health and integrity of the environment by implementing this comprehensive framework.

### 10.1 Urgent Call for Interdisciplinary Action

**Figure 7:**
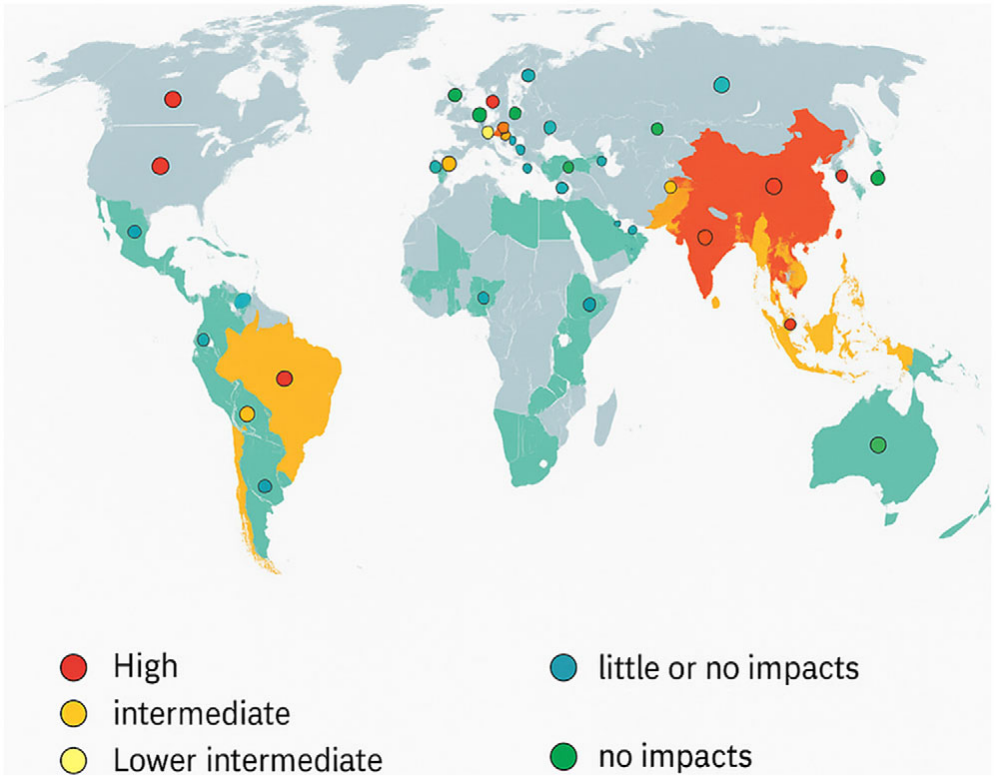
Global distribution of pigment‐driven hazards informed by environmental and health case studies, pigment production tonnage, and regulatory frameworks by 2021.^[^ ^104^ ^]^ Color‐coded markers indicate regional significance, red for high, yellow and orange for intermediate levels of case studies, and blue and green for places that have little or no impacts and more advanced regulatory policies. This visualization highlights disparities in pigment regulation and the need for global harmonization of safety standards. Arrow numbers exhibit increasing tonnage of production across globe (image drawn with Biorender and SORA).

Scientists, policymakers, and industrialists need to act swiftly and co‐ordinately to address pigment contamination and usage challenges. As illustrated in **Figure** **7**, there is an urgent need for globally harmonized safety standards, since disparities in pigment regulation and risk exposure reveal uneven environmental and health burdens across regions with high production. To enable this design, team strengthening should be emphasized through interdisciplinary integration consisting of chemists, toxicologists, ecologists, and public health professionals and policymakers. Progressing policy frameworks is also key, especially the internationally aligned policies for production, use, and disposal of pigments to mitigate risks, particularly in at‐risk areas.^[^ ^92^ ^]^ Community engagement also supports other best practice approaches that aim to raise public awareness, enable stakeholder participation, and promote sustainability so that decisions are informed and balanced. Proactive governance, continuous adaptation of regulatory frameworks, and fostering green innovation are paramount to effectively managing these complex materials. A strong, forward‐looking conclusion ensures the article's message resonates and encourages further engagement with the proposed solutions.

### 10.2 Vision for a Sustainable Pigment Future under One Health

Focusing on One Health will shape the future of pigments to be both sustainable and innovative. This vision includes green innovation aimed at designing pigments with low environmental and health risks using biodegradable, nontoxic alternatives.^[^ ^103^ ^]^ It also highlights the importance of strong risk assessment, with the development of advanced analytical tools and methodologies to enable effective monitoring and management of pigment‐related risks. Moreover, a global commitment is also needed that encourages countries to implement integrated strategies following the One Health concept for the fair and sustainable management of pigments worldwide. This will lead to a future of pigments that ease human, animal, and environmental health through interdisciplinary collaboration, better global policies, and new technology development.

## Conflict of Interest

The authors declare no conflict of interest.
